# Hughes-Stovin Syndrome

**DOI:** 10.1186/1750-1172-6-15

**Published:** 2011-04-13

**Authors:** Umair Khalid, Taimur Saleem

**Affiliations:** 1 Medical College, Aga Khan University, Stadium Road, Karachi 74800, Pakistan

## Abstract

Hughes-Stovin Syndrome (HSS) is a very rare clinical disorder characterized by thrombophlebitis and multiple pulmonary and/or bronchial aneurysms. Less than 40 published cases of HSS have been described in English medical literature so far. The exact etiology and pathogenesis of HSS is unknown; possible causes include infections and angiodysplasia. HSS has also been considered as a variant of Behcet's disease (BD). Patients with HSS usually present with cough, dyspnea, fever, chest pain and haemoptysis. The management of HSS can either be medical or surgical. Medical management includes the use of steroids and cytotoxic agents. Cyclophosphamide, in particular, is a favored therapeutic agent in this regard. Antibiotics have no proven role in HSS while anticoagulants and thombolytic agents are generally contraindicated due to an increased risk of fatal hemorrhage. However, their use may be considered with great care under special circumstances, for instance, intracardiac thrombi or massive pulmonary embolism. For cases of massive hemoptysis due to large pulmonary aneurysms or those with lesions confined to one segment or one lung, lobectomy or pneumectomy can be carried out. However, surgical risks merit serious consideration and must be discussed with the patient. Transcatheter arterial embolization has emerged as a less invasive alternative to surgery in selected cases of HSS. Overall, patients with HSS have a poor prognosis and aneurysmal rupture is the leading cause of death. However, early diagnosis and timely intervention is crucial in improving the prognosis. There is a need to clearly elucidate the genetic, etiologic and pathologic basis for HSS in the future. Although most of the evidence put forward to refute the role of an infectious agent in the etiology and pathogenesis of HSS is based on negative blood and other body fluid cultures, more robust objective assessment is needed through the use of electron microscopy or 16 sRNA studies. The development of better therapeutic agents is also needed to address and prevent the serious consequences arising from pulmonary arterial aneurysms seen in BD and HSS. Also, the issue of anticoagulation in these patients is challenging and requires further deliberation.

## Disease name and synonyms

Hughes-Stovin syndrome (HSS, ORPHA228116) was named after two British physicians, Drs. John Patterson Hughes and Peter George Ingle Stovin. They first described the findings of the syndrome (deep venous thrombosis and segmental pulmonary artery aneurysms) in a total of four male patients with pulmonary artery aneurysms in 1959 [1]. Two of these patients were their own while the remaining two had been described previously in literature [2]. The syndrome has not been referred to by any other synonym in medical literature.

## Definition and diagnostic criteria

HSS is a rare disorder of unknown etiology. Although the association between multiple pulmonary artery aneurysms and venous thrombosis of the lower limbs had been reported by Beattie and Hall in 1911, it was not until 1962 that the eponym "Hughes-Stovin Syndrome" was formally introduced in medical literature for HSS [2].

Being an extremely rare disease, there is no formally described diagnostic criteria or pathognomonic laboratory investigation for this syndrome. Generally, the syndrome is characterized by the findings of thrombophlebitis and multiple pulmonary and/or bronchial aneurysms [3]. One other syndrome, Behcet's disease (BD), is also associated with this aneurysm-thrombosis combination.

Turkish venercologist Halushi Behcet described the constellation of hypopyon, iritis and orogential ulcers in 1937 [4]. However, a decade earlier, Adamantiades had reported the disease as well. Although this gave birth to the eponym Adamantiades-Behcet's disease [5-7], the syndrome continues to be widely referred to as BD in medical literature. The acronym MAGIC describes the features of "Mouth And Genital ulcers with Inflamed Cartilage" seen in BD [7].

Therefore, if a patient presents with this set of findings (aneurysms and thrombosis) and the clinician is able to rule out other causes, then the patient either has HSS or BD. However, BD can be ruled out if its other distinctive features are absent in the patient. This is how HSS has been diagnosed in the majority of the case reports in literature.

## Epidemiology

HSS is an exceedingly rare disorder with less than 40 published cases in English medical literature. For this reason, its population-based incidence can't be determined. It usually affects the young adult population bracket (reported cases ranged in age from 12 to 48 years) and holds a strong predilection for the male gender [8-10]. HSS does not appear to have preponderance for any geographic location. Cases of HSS have been reported from diverse geographical areas including North America, Europe, Africa and Asia [11-14]. None of the reports have mentioned consanguineous marriages in the parents of the patients suffering from HSS. Thus, the genetic basis and familial predisposition of HSS remains nebulous.

## Clinical description

About 25% of patients with HSS develop thromboembolism, arterial aneurysms and vascular occlusions. The distribution of the vascular component of the syndrome is as follows: arterial (7%), venous (25%) or both (68%) [15]. The clinical paradigm of HSS can be divided into three phases [3,10]:

a. Symptoms of thrombophlebitis

b. Formation of large pulmonary and/or bronchial aneurysms

c. Aneurysmal rupture leading to massive hemoptysis and death

**Figure 1:**
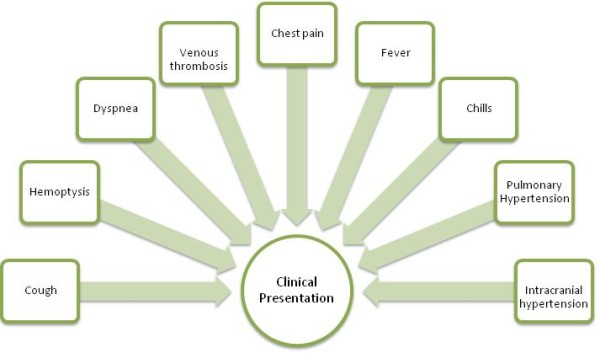
**Signs and symptoms of Hughes-Stovin Syndrome**.

These stages usually evolve successively. The first and second phases have to be present for the diagnosis of HSS while the third phase is the usual ultimate outcome for untreated patients. The typical presenting features of HSS are related to the presence of the pulmonary aneurysms and peripheral venous thrombosis. These signs and symptoms are listed in **figure **1[8,16-18]. Patients can have seizures, diplopia and cephalalgia secondary to raised intracranial pressure consequent to cerebral venous sinus thrombosis [1,19]. The raised intracranial pressure also accounts for the papilledema of the optic disc observed in some patients with HSS [20].

Aneurysms observed in HSS maybe single, multiple, unilateral or bilateral [15]. These aneurysms generally involve the pulmonary and bronchial arteries but can also occur anywhere in systemic circulation. For example, Herb et al have reported HSS in a patient who had an aneurysm of the left hepatic artery [21]. Similarly, aneurysms in the iliac artery have also been described [20]. The low-pressure aneurysms are usually benign with a minimal risk for vessel dissection or rupture [22]. Even in the cases of large dilatations with diameters up to 16 cm, this risk remains low as long as the vascular pressures remain within control. On the other hand, high pressure aneurysms are associated with serious morbidity and mortality [23].

Recurrent phlebitis frequently involves the large vessels resulting in thromboembolism, with even reports of thrombosis of the vena cava, cardiac chambers, jugular vein, iliac vein, femoral vein and dural sinuses [8,10,16,19,24-27]. Patients with thrombosis in the vena cava may have engorged abdominal superficial veins [26].

## Etiology and pathogenesis

The exact etiology and pathogenesis of HSS is currently unknown. Several proposed theories have attempted to explain the manifestations of this rare entity [16,28]. The current consensus is that vasculitis is the primary pathologic process underlying HSS [8].

### 1. Infections

Septic embolisms and abscesses have been proposed as the cause of pulmonary aneurysms by some authorities [3,29-31]. In support of this theory, pulmonary aneurysms have been preceded by infections in some cases, including scrotal abscesses [1,29], epididymitis [2] and oophoritis [32]. Venous thrombosis can also be considered to arise as a consequence of septic emboli, bacterial toxins or hyperergic reactions [33].

However, infectious agents, as an etiology for HSS, have generally received less attention due to two reasons. Firstly, various antibiotic regimens that have been tried in the treatment of HSS have proven ineffective. Secondly, there has been a lack of positive blood cultures in the evaluation of patients with HSS [1-3,11,29,31,32,34,35]. Cultures of other body fluids in patients have also been found to be sterile [2]. Nevertheless, the possibility of undetected organisms of low-grade virulence has been speculated [12]. According to the initial hypothesis [1], pulmonary artery aneurysms may develop from a degenerative defect in the bronchial arteries or may even be mycotic in origin resulting from emboli infected with low-grade virulence organisms. However, subsequent studies have failed to find such comparable defects in the bronchial arteries.

For BD, the following infectious agents have been implicated in the pathogenesis but never conclusively proven: Hepatitis A, B, C, E viruses, Herpes Simplex Virus (HSV), Parvovirus B19, *Helicobacter pylori*, *Chlamydia pneumoniae*, *Streptococcus sanguis, Streptococcus mitis, Streptococcus salivarius *and *Saccharomyces cerevisiae *[36]. The details for these agents in the pathogenesis of BD have been given in **table **1. Interestingly, in support of the infectious etiology of BD, it has been suggested that proximity to the Silk Road and interactive trade activities may have provided the necessary bridge for the extension of the disease between the Mediterranean and the East [5].

**Table 1:** Infectious agents implicated in the pathogenesis of Behcet's disease (adapted from Mendoza-Pinto et al [5] and Kapsimali et al [36])

Agent(s)	Pertinent rationale or refutation for involvement in Behcet's disease
Hepatitis A, B, C, E viruses	- Serological evidence of previous HAV, HCV and HEV infections not significantly different in patients with Behcet's disease as compared to controls. - Previous HBV infection, however, seen in a significantly lower number of patients with Behcet's disease as compared with healthy controls.

Herpes simplex virus (HSV)	Anti-HSV-1 antibodies observed more commonly in patients with Behcet's disease than controls. -DNA of HSV detectable in genital and intestinal ulcers but not in oral aphthous ulcers.

Parovirus B19	Parvovirus B19 IgG antibodies reported more in patients with Behcet's disease as compared to controls.

*Helicobacter pylori*	Almost equal proportion of patients with Behcet's disease and controls had *H. pylori *infection following eradication therapy.

*Chlamydia pneumoniae*	- IgG seropositivity for *C. pneumoniae *between cases and controls not significantly different. - However, proportion of seropositive cases with higher IgG titres was greater.

*Streptococcus sanguis, Streptococcus mitis *and *Streptococcus salivarius*	- Attenuation of skin and arthritic involvement in Behcet's disease after antibiotic administration. - Hypersensitivity to cutaneous streptococcal antigens reported. - Aggravation of symptoms after dental manipulations. - Treatment of chronic oral infections impacts long term prognosis of disease positively.

*Saccharomyces cerevisiae*	Unclear role, distribution and pathogenetic relationship of ASCA antibodies in patients with Behcet's disease.

*Heat shock proteins*	- Role for heat shock proteins of mycobacteria and streptococci suggested in Behcet's disease. - Model of molecular mimicry thought to be responsible for manifestations of Behcet's disease.

### 2. Angiodysplasia

Angiodysplasia of bronchial arteries is another debatable hypothesis to account for the vascular changes [16,28,37]. Hughes and Stovin suggested that the structural changes in the bronchial arteries impaired the provision of adequate nutrition to the pulmonary arteries through the vasa vasorum. In turn, these events led to inflammation, damage to the elastic tissue and creation of arterial aneurysms [21]. Conversely, it has been suggested that the occlusion of the pulmonary arteries causes increased flow and pressure in the bronchial arteries which predisposes to the formation of bronchial artery aneurysms [21].

### 3. Possible manifestation of Behcet's Syndrome

Some authors have suggested that HSS may actually be a partially manifested BD owing to their similar findings instead of a novel syndrome [16,19,38,39]. In fact, HSS and BD are the only vasculitides associated with the development of pulmonary artery aneurysms [4,40,41]. HSS has been variably described as "the cardiovascular manifestation of Behcet's disease" [14], "incomplete Behcet's" [19] and "a rare case of Behcet's disease" [42] in literature.

### 4. Mechanism of Thrombosis

The pathogenesis of thrombosis remains unclear in both HSS and BD. Thrombophilia is not believed to be a major contributory factor to the development of thrombosis in these patients [15]. Possible mechanisms to explain the prothrombotic nature of BD in literature include: progressive decline in endothelial progenitor cells, direct endothelial injury, aberrant fibrinogenolysis and platelet activity, abnormal levels and expression of thrombomodulin, adrenomodulin and vascular endothelial growth factor (VEGF), E-selectin activation and variable nitric oxide levels [5,36]. Some reports have also described the contribution of prothrombin gene mutations and aberrant protein C levels and activity in the thrombotic events of BD [43,44].

The presence of hyperhomocyteinemia independently adds to the risk of venous thromboembolism in HSS. Homocysteine causes thrombosis through multiple mechanisms including the activation of platelets, increased thrombin formation, impairment of fibrinolysis and endothelial dysfunction through lipid peroxidation and endothelial injury [45].

An important detail that merits consideration here is that the clot in the pulmonary arteries in HSS or BD arises mostly due to the arterial vasculitis rather than venous thromboembolism, especially in patients without deep venous thrombosis. Also, the thrombin in the lower extremities is tightly adherent to the inflamed veins in BD and HSS patients [12,19,37]. Balci et al [20] have reported the case of a patient in whom the pulmonary emboli recurred despite the placement of the Greenfield filter. Although the event can simply be attributed to the failure of the Greenfield filter, the possibility of the in situ formation of the pulmonary embolus as a separate entity from the deep venous thrombus can't be ignored.

### 5. Extrapolation of the pathologic model for Behcet's disease

As mentioned earlier, although the exact pathologic basis for HSS is unclear, it may be similar to the model for BD as the two conditions share many features. Interplay of multiple factors such as genetic, environmental, immunological and endothelial is most likely to be involved in the pathogenesis of BD [36].

Strictly speaking, BD is not considered an autoimmune disease [36] because of the following reasons: a) B-cell hyper-reactivity not noted, b) female predominance not reported across the board, c) absence of Sjogren syndrome in patients with BD. However, immunologic mechanisms, different from those in other autoimmune diseases, are believed to be involved in the pathogenesis of BD. A growing body of evidence is suggestive of the active role of T-cell mediated immune mechanisms and responses in BD. In particular, γδ+T-cells are thought to be involved in BD and their stimulation results from microbial antigens produced by the oral flora [36]. The exact role and interactions of CD4+ T-cells and its subsets, CD8+ T-cells, double negative T-cells and other antigen presenting cells (APCs) in BD are also being investigated. In addition, neutrophil hyperactivation, a key component of inflammatory vasculitis seen in BD, occurs secondary to the release of the battery of cytokines from the APCs and T-cells. Neutrophils exhibit increased generation of reactive oxygen species (ROS), phagocytic capacity and cytokine production as well [5,36].

The main players of the cytokine axis active in BD include interferon-γ, IL-1b, IL-6, IL-8, IL-12, IL-18 and TNF-α [5,36]. A role of autoantibodies in the expression and manifestations of BD has also been proposed (**table **2) [5,36].

**Table 2:** Autoantibodies proposed to be involved in pathogenesis of Behcet's disease (adapted from Mendoza-Pinto et al [5] and Kapsimali et al [36])

#	Autoantibody
1.	Anti-endothelial antibody (α-Enolase autoantibody)

2.	Antineutrophilic cytoplasmic antibody (ANCA)

3.	Anticardiolipin antibody

4.	Autoantibody to Retinal S antigen

5.	α-Tropomyosin autoantibody

6.	Kinectin autoantibody

### 6. Bridge to immunogenetics

Classically and most convincingly, the association of BD has been described with the human leukocyte antigen (HLA) B51 in literature. In a recent met-analysis/systematic review comprising 4,800 cases and 16,289 controls, the pooled odd's ratio for the susceptibility to BD associated with HLA-B51/B5 carriage was 5.78 (95% CI: 5.00 - 6.67). The population-attributable risk (PAR) of HLA-B51/B5 in relation to BD was 32% (Northern/Eastern Europe) to 52% (Southern Europe). This study also showed that the random-effects pooled prevalence for HLA-B51/B5 was 57.2% (95% CI: 53.4 - 60.9%) in cases of BD versus 18.1% (95% CI: 16.1 - 20.3%) in controls [46]. It is speculated that HLA-B51 forms an integral part of an immunologic axis in patients with BD that interacts with cross-reacting self-antigens and immunoglobulin-like receptors on immune cells of the body [36]. However, it is still unclear whether the strong association of BD with HLA-B51/B5 is representative of a true causal association or demonstrates linkage disequilibrium with another gene that is operational in BD [46].

In addition to the strong association of BD with alleles of the major histocomptability complex (MHC), recent studies have also highlighted the polygenic status of BD and investigated the role of additional genes in BD including, but not limited to MIC, MEFV, TNF, HSP etc [5,36].

In contrast, out of all the literature reviewed, only one report described the testing of a patient with HSS [13] for HLA-B51. The latter was found positive in this patient. However, the trend obviously needs to be confirmed in other patients before deriving any conclusions. Also, it is not known whether patients in other reports on HSS were tested for HLA-B51 or not as no specific comment regarding such testing was made or alluded to in those reports.

## Diagnostic considerations

The diagnosis of HSS can be difficult owing to a non-specific set of findings.

### 1. Laboratory findings

The laboratory findings in HSS patients are non-specific. The patients can have leukocytosis, anemia, raised erythrocyte sedimentation rate (ESR) and elevated C-reactive protein (CRP). Authors have also used the following tests in their assessment of HSS patients at initial presentation to rule out a battery of competing diagnosis: coagulation studies, anti-nuclear antibody, rheumatoid factor (RF), serum complement levels (C3 and C4), anti-double stranded DNA antibody, anti-neutrophil cytoplasmic antibodies (c-ANCA and p-ANCA), anti-cardiolipin antibodies, antistreptolysin O titres, hepatitis viral serology, hemoglobin electrophoresis, examination of bronchial washings, serological tests for detecting infection by *Treponema pallidum *and HIV and bone marrow biopsy [2,8,15,21,28,38,41,45].

### 2. Bronchoscopy

Bronchoscopy is often done in HSS patients who present with hemoptysis. In patients with bronchial artery aneurysms, fibre bronchoscopy can show pulsatile tumor(s) with fibrinoid onlayers and/or ectatic vessels [21] or bronchial obstruction caused by submucosal mass [38].

### 3. Ventilation-perfusion (V-Q) scan

Patients with HSS can develop pulmonary embolism. The formation of these emboli has been attributed to the inflammatory response of the endothelial cells lining the vessels [26]. The V-Q scan in such patients shows area(s) with ventilation-perfusion mismatch.

### 4. Doppler ultrasound of extremities

Peripheral venous thrombosis is an important part of HSS. Color Doppler examination of the extremities should, therefore, be undertaken to evaluate the presence of deep venous thrombosis on the basis of reasonable clinical suspicion.

### 5. Radiological diagnosis

#### a. Chest roentograms

**Figure 2:**
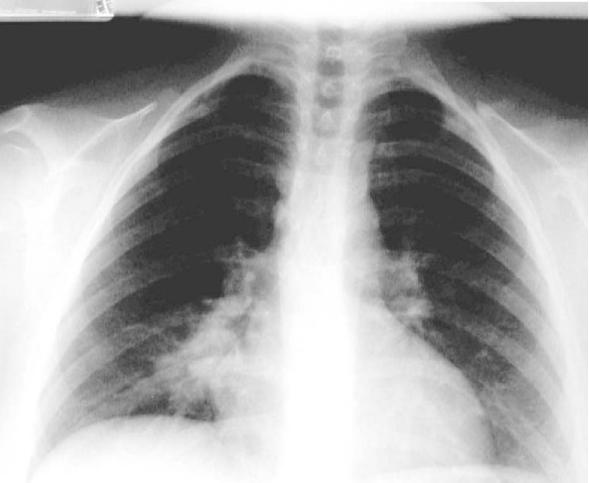
**X-ray of the chest showing an infiltrate in the lower lobe of the right lung**. Reproduced with permission from Al-Jahdali H [15]

**Figure 3:**
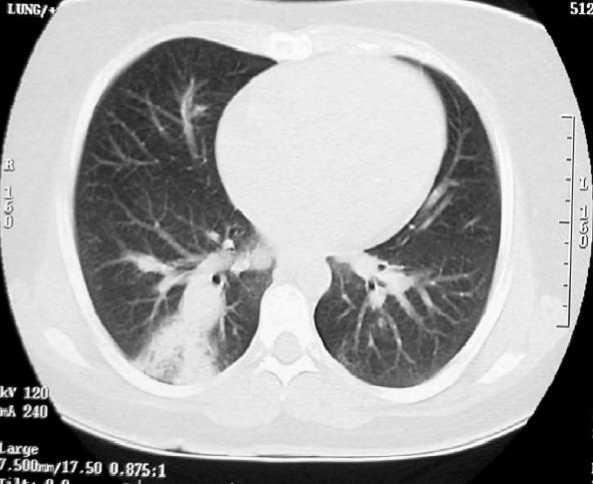
**CT scan of the chest showing ill defined infiltrate in lower lobe of the right lung**. Reproduced with permission from Al-Jahdali H [15].

Chest roentgograms (**figure **2 and 3) depict pulmonary artery aneurysms as hilar enlargements or round, lobulated opacities [47].

#### b. Conventional angiography

**Figure 4:**
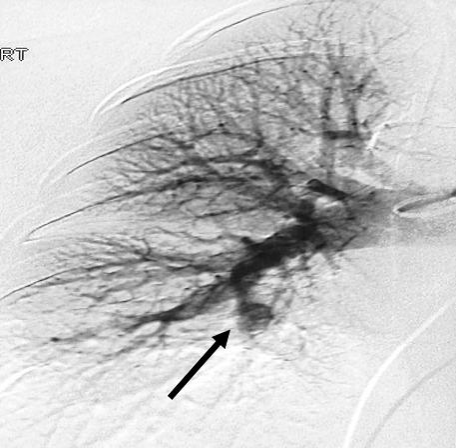
**Pulmonary angiography showing an aneurysm of the interlobar pulmonary artery**. Reproduced with permission from Al-Jahdali H [15].

Traditionally, conventional angiography (**figure **4) has been regarded as a gold standard for the diagnosis of pulmonary artery aneurysms. It also aids in assessment of angiodysplastic bronchial arteries in HSS. The characteristic picture seen is aneurysmal formation proximal to the occluded segments while distal to the interruption, signs of hypoperfusion are observed [48]. However, it should be noted that selective pulmonary angiography can be hazardous as it carries the risk of aneurysm rupture [17].

#### c. Helical computed tomography

**Figure 5:**
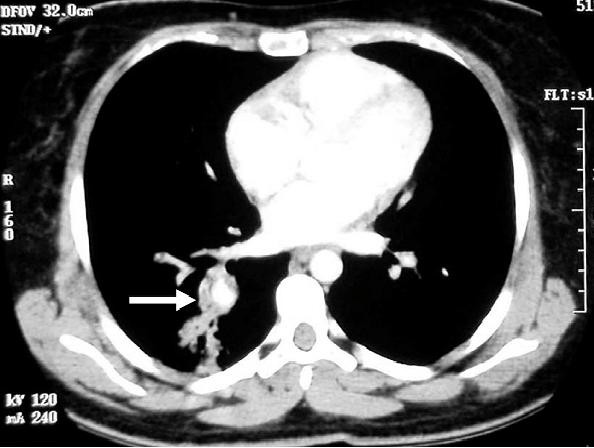
**Contrast enhanced CT scan of chest showing pulmonary artery aneurysm**. Reproduced with permission from Al-Jahdali H [15].

However, it may not be possible to perform angiography in all patients; especially in patients with thromboses in the vena cava which limit the passage of the catheter [26]. In such cases, other non-invasive modalities such as helical computed tomography (CT) may demonstrate high quality vascular images with minimal amount of contrast material used (**figure **5). In fact, multi-detector row helical CT angiography now offers more precise visualization of large systemic arteries than does conventional angiography. As such, it can be regarded as an emerging and effective standard in the diagnosis of pulmonary artery aneurysms because of its non-invasive nature, ease of performance and increasing availability. Furthermore, Ketchum et al showed that 3D volume rendering analysis can detect morphologically abnormal, tortuous branches of bronchial arteries even before aneurysm formation [12,49]. Mahlo et al [17] and Herb et al [21] recorded distorted and dilated bronchial arteries with convoluted small branches when they performed digital subtraction angiography of the bronchial arteries.

#### d. Magnetic resonance angiography

Magnetic resonance angiography (MRA) is relatively less sensitive than helical CT for picking up small aneurysms [20,26,47,49].

### 6. Histological diagnosis

Classic histopathologic findings of HSS [1,2] include diffuse dilatation and partial occlusion of the aneurysmal arteries, perivascular infiltration that is predominantly lympho-monocytic and diffuse proliferative sclerosis. The elastic and muscular fibers become annihilated whereas tunica media is completely filled with lymphocytes, plasma cells and foam cells in the affected vessels. The perivascular infiltrate extends into the adventia and into the overlying thrombus. Macrophages containing hemosiderin may also be observed. In the pulmonary veins, fibrosis and muscular medial thickening is seen. However, Durieux et al [37] described a dense neutrophilic infiltrate in the walls of the vessels in HSS. On the other hand, Meireles et al [35] reported the histologic findings of HSS in a patient's necropsy as medial hypertrophy, intimal fibrosis with marked eosinophilic infiltrates.

In comparison, the histology of vascular lesions in BD is characterized by a triad of "neutrophilic infiltration", "endothelial cell swelling" and "fibrinoid necrosis" [5]. Similarly, neutrophils establish an early presence in the mucocutaneous and ocular lesions of BD [5].

The pulmonary arteries showed widespread lesions of Regardless of the modality used, a complete visualization of prominent bronchial arteries and pathologic enhancement of pulmonary thromboembolism should alert physicians to the suspicion of pulmonary vasculitis. A timely diagnosis and intervention is imperative to prevent potentially life-threatening massive hemorrhage due to pulmonary aneurysms [12].

## Differential diagnosis

Amongst the causes for pulmonary artery aneurysm (**table **3) [50], there are two idiopathic, albeit similar, syndromes that are associated with thrombosis: BD and HSS. There exists a significant overlap between the clinical, radiological and histopathological findings of HSS and BD (**table **4). Specifically, pulmonary involvement is often indistinguishable between the two entities. However, whether the two conditions are identical is open to both debate and speculation as the exact pathophysiology of both syndromes remains unclear.

**Table 3:** Causes for pulmonary artery aneurysms without arteriovenous communication (adapted from Fischer et al [18])

1.	Infection
	Tuberculosis (Rasmussen's aneurysms)

	Syphilitic

	Other (bacterial and fungal); may arise from right sided endocarditis

2.	**Structural cardiac abnormalities**

	Congenital heart disease

	Acquired cardiac abnormalities

	Structural vascular abnormalities

	Congenital

	Cystic medionecrosis/atherosclerosis

	Acquired

	Marfan's syndrome

	Behçet's disease

3.	**Pulmonary hypertension**

4.	**Idiopathic vasculitic syndromes**

	Hughes-Stovin syndrome

	Behçet's disease

5.	**Trauma **(for example, from a Swan-Ganz catheter)

6.	**Miscellaneous**

**Table 4:** Similarities in pulmonary involvement between Behcet's disease and HSS (adapted from Erkan et al [38])

Characteristic	Details
Gender	Predominantly young males

Triad of clinical findings	Fever, arthalgias, thrombosis

Occurrence of thrombosis with pulmonary artery aneurysms	HSS - 100%; Behcet's disease - 80%

Overlapping histopathologic features	Destruction of arterial walls, perivascular infiltrates

Therapy	Cytotoxic drugs and corticosteroids

Most common cause of death	Rupture of pulmonary artery aneurysm

The target populations as well as the pulmonary manifestations in BD appear to be similar to that in HSS (**table **4). Also, both diseases are characterized histologically by the destruction of the vessel walls of pulmonary vasculature along with perivascular infiltration [37,50]. BD primarily affects young adults, especially males [40,41,51]. However, this gender distribution is not universal. There are also studies which have shown a female preponderance [52,53]. This is in contrast to HSS where the majority of the cases (>80 - 90%) have been seen in male population. Although BD is found all over the world, certain regions like Far East, the Mediterranean (the ancient "Silk Road") and the Middle East have reported higher rates of prevalence [36,40,41,51,54]. BD has been known to occur more commonly in geographic areas that fall between latitudes 30 and 45 degrees north [7]. The incidence of BD in different regions is as follows: North America and Europe - 0.38 - 7.5/100,000 and Turkey - upto 42/100,000 [36].

Findings unique to BD are recurrent genital ulceration, eye lesions, skin lesions, iritis, arthralgia and a positive pathergy test [40,55,56] and this helps in distinguishing the two entities. For the clinical diagnosis of BD, the patient must have recurrent oral ulceration with atleast two of the following clinical manifestations: recurrent genital ulceration, skin lesions, eye lesions or a positive pathergy test [57]. The pathergy test is performed by taking a sterile 20 - 22 gauge needle and obliquely piercing the skin to a depth of 5 mm. If the site develops an erythematous papule after 48 hours, the test is positive [5].

It is believed that the aneurysms seen in BD arise either due to the process of obliterative endarteritis of the vasa vasorum or they are pseudo-aneurysms characterized by edematous vessel walls. The latter are usually formed after perforation [38].

## Management

Owing to the lack of controlled trials, there are no standard treatment guidelines for the management of HSS. As BD and HSS share certain clinical characteristics and manifestations, the management of HSS can be tailored along the lines of BD [19]. Despite this, it should be noted that the European League Against Rheumatism (EULAR) has acknowledged the need for properly designed and robust prospective studies for improving management strategies even for BD [58,59]. In this section, relevant EULAR recommendations with regards to the management of BD disease have been referred to, especially with regards to vascular disease.

### 1. Medical management

#### a. Immunosuppressive therapy

Most commonly, immunosuppressive therapy involving a combination regimen of glucocorticoids and cyclophosphamide has been employed as a first line medical management in the treatment of HSS, although its effectiveness remains to be fully established [8,40]. The steroids are usually administered as pulse IV therapy followed by oral steroids usually with subsequent taper [15,19,38,45]. Depending upon the clinical response, steroids can be discontinued but cyclophosphamide is usually given for at least one year after complete remission [4]. One described regimen for the treatment of arterial aneurysms is monthly pulses of cyclophosphamide (1 gram) plus prednisolone (1 mg/kg/day). The latter is then tapered over the course of several months to a dose less than 30 mg/day [60].

Immunosuppression has the potential to stabilize small aneurysms in the pulmonary vasculature [8], and in some cases can even make them regress [27]. Other agents that have been variably used in the treatment of HSS include colcichine, cyclosporine and azathioprine [8,15,38]. However, despite the favorable response seen in some cases, the caveat that needs to be remembered is that immunosuppressive therapy may not always be helpful in the cessation of disease progression especially if the disease has already evolved to an advanced stage [61].

EULAR recommends the use of steroids, azathioprine, cyclophosphamide or cyclosporine A for the management of acute deep vein thrombosis of BD while cyclophosphamide and corticosteroids have been recommended for pulmonary and peripheral arterial aneurysms in BD [58]. For pulmonary aneurysms, EULAR has recommended the continued use of cyclophosphamide for two years followed by azathioprine [58]. Cyclosporine A, being neurotoxic, should not be used in patients with neurological manifestations of BD [58].

#### b. Antibiotics

Antibiotics have no proven role in the management of HSS [1,2,29,34].

#### c. Anticoagulants and thrombolytic agents

Anticoagulants and thombolytic agents are generally considered contraindicated due to an increased risk of fatal hemorrhage, even though they confer a beneficial effect in an embolic state [37]. Some patients with HSS already have hemoptysis at initial presentation; making these agents an unsafe therapeutic option. This places physicians on the horns of a dilemma because of the pro-thrombotic nature of the syndrome and the occurrence of potentially life-threatening events such as intracardiac thrombi or pulmonary embolism. Therefore, anticoagulation may be employed with great vigilance in a few carefully evaluated circumstances where the benefits are believed to significantly outweigh the risks. Kim et al successfully used anticoagulation in a patient with HSS, hyperhomocysteinemia and intracardiac thrombi employing warfarin with enoxaparin as a bridge [45]. Tsai et al have recommended that in patients with pulmonary embolism and HSS, anticoagulation should be used very judiciously. This is only in patients with embolisms in the main pulmonary artery that lead to life-threatening clinical deterioration and hemodynamic instability [26]. Anticoagulation maybe used to prevent or treat deep vein thrombosis after the pulmonary artery aneurysms have been surgically resected [15] or after adequate immunosuppressive treatment has been given [40]. This is usually achieved by starting the patient on intravenous or subcutaneous heparin and then shifting to oral warfarin therapy. However, the caveat here is that patients may still develop thrombosis despite adequate anticoagulation [38]. Another point to consider is the possible role of deep venous thrombi in aneurysm formation. By that logic, long term anticoagulation may have the potential to prevent further aneurysm formation by targeting this particular mechanism [62].

The issue of anticoagulation in patients with HSS and BD is obviously complex and requires focused studies before any definite recommendation can be made. EULAR has stressed the need for controlled trials to evaluate the utility of anticoagulation in patients with BD. The use of anticoagulants and antifibrinolytic agents in BD is not currently recommended by EULAR [58].

#### d. Antiplatelet agents

In the absence of extensive thrombi, some authors have suggested the use of antiplatelet agents such as low dose aspirin in patients [63,64]. However, EULAR doesn't currently recommend the use of antiplatelet agents in BD [58].

#### e. Ventilator support

Patients presenting with severe hemoptysis may require initiation of mechanical ventilator support [65].

#### f. Surgical consultation

Along with the initiation of medical management, an urgent consultation should be given to the cardiothoracic surgery team, especially if the patient presents with severe or recalcitrant hemoptysis [26].

### 2. Surgical management

For the cases of massive hemoptysis due to large pulmonary aneurysms or those with lesions confined to one segment or one lung, lobectomy or pneumectomy can be carried out to remove the aneurysms based on the data from published case reports. Kindermann et al have described the resection of pulmonary artery aneurysm with the reconstruction of the arterial segment using a saphenous vein grafts [66]. Durieux et al [37] used surgical intervention for three cases of HSS who had isolated pulmonary aneurysms and none of them showed any signs of recurrence on subsequent follow ups. However, this is not the case for most patients of HSS where bilateral, extensive pulmonary aneurysms limit the role of surgery as the frontline treatment modality. Furthermore, high operative morbidity and mortality associated with surgery is another consideration that must be discussed with patients [1,2,66,67]. Additionally, after surgery, there is a 25% risk of recurrence of aneurysms at the site of anastomosis [68].

For BD, Alexoudi et al have recommended that surgery be considered as a treatment of choice for vascular involvement in the following circumstances: expanding aneurysm, acute rupture and severe ischemia [69]. However, surgeons should be aware of the possibility of the formation of pseudoaneurysms (if arterial involvement) and false anastomotic aneurysms (if venous involvement) after surgical intervention. The pre-operative use of doxycycline has been recommended because of its potential role in off-setting these deleterious post-operative events [69].

### 3. Transcatheter arterial embolization

For patients who are not suitable candidates for aggressive surgical intervention, transcatheter arterial embolization, being a less invasive procedure, offers a suitable and effective alternative in HSS [8]. Furthermore, since aneurysms in HSS are usually bilateral and multifocal at the time of diagnosis, embolization is a preferred modality in such patients [8]. Arterial embolization is also an acceptable therapeutic option in patients with severe or recurrent hemoptysis [21]. Authors have performed embolization with several agents including steel coils, ethibloc and an epoxy, isobutyl cyanoacrylate [8]. Associated complications of arterial embolization include arteriovenous fistulae, pulmonary infarction, abscess formation, oesophageal necrosis, bronchial necrosis, and spinal ischemia [70,71]. Rarely, the patients may require repeat embolization because the arterial lesions may become recanalized or revascularized [21]. Balloon venoplasty may be used in patients with vena caval thrombosis. However, it is only safe to perform this procedure in the early stages [72].

## Prognosis

Aneurysms of arterial origin portend a poorer prognostication than venular aneurysms [15]. In particular, pulmonary artery aneurysms have a poor prognosis. Hemoptysis can arise from three mechanisms in patients:

1. Aneurysmal rupture; it is the leading cause of death in patients of HSS. The erosion of the ruptured aneurysm into a bronchus leads to hemoptysis [73].

2. Active vasculitis that can lead to thrombosis [73].

3. Bronchial artery hypertrophy secondary to ischemia that, in turn, has been attributed to the pulmonary artery occlusion [21]. Mahlo et al. speculated that the cause of death in HSS could be due to the rupture of angiodysplastic bronchial arteries rather than rupture of aneurysmal pulmonary arteries [17].

Early diagnosis and timely intervention is, therefore, crucial in improving the prognosis of patients with HSS. Appropriate treatment, if instituted promptly and early in the course of the disease, has the potential to induce remission [4,40,47,54,74].

## Future directions

There is a lack of clear diagnostic criteria and management guidelines for HSS. The disease is clearly a rare but grave clinical entity and has not been extensively studied so far. Most of the data on HSS is in the form of sporadic case reports. Establishing diagnostic criteria and formulating management guidelines for HSS is imperative to standardize the quality of care delivered and to improve prognosis across the different geographic regions of the world since HSS is associated with significant morbidity and mortality. There is also a need to better elucidate the genetic basis and familial preponderance, if any, of HSS. As with any genetic disease, knowledge of the latter will be helpful to clinicians in the provision of pre-conception genetic counseling to patients with HSS.

Future investigations should include human leukocyte antigen (HLA) typing in patients to compare and contrast the genetic basis of HSS with BD. Although it should be acknowledged that HSS and BD share many clinical, radiological and histological features, the suggestion that they are, in essence, the same disease can neither be currently accepted nor categorically refuted because the pathologic, genetic and etiologic basis of both conditions has not been clearly unraveled. These aspects have been highlighted for focused research in the future. Unless these arenas are elucidated, the "pathogenic kinship" between the two conditions remains obscure [18] and should be examined with scientific skepticism and perspicacity. Although most of the evidence put forward to refute the role of an infectious agent in the etiology and pathogenesis of HSS is based on negative blood and other body fluid cultures, more robust objective assessment is clearly needed through the use of electron microscopy or 16 sRNA studies. Finally, the development of better therapeutic agents is needed to address and prevent the serious consequences arising from pulmonary arterial aneurysms seen in BD and HSS. Also, the issue of anticoagulation in these patients is challenging and requires further deliberation.

## Competing interests

The authors declare that they have no competing interests.

## Authors' contributions

UK performed the literature search, interpreted the data and drafted the manuscript. TS conceived the project, performed the literature search, interpreted the data, drafted the manuscript and critically revised it. All authors have read and approved the final manuscript.
